# An Enigmatic Case of a Febrile Infant With Seizures

**DOI:** 10.7759/cureus.25663

**Published:** 2022-06-04

**Authors:** Bhavishya Devireddy, Whitney Kalin, Frederick Laningham, Fouzia Naeem

**Affiliations:** 1 Pediatrics, Valley Children’s Healthcare, Fresno, USA; 2 Radiology, Valley Children’s Healthcare, Fresno, USA; 3 Pediatric Infectious Diseases, Valley Children’s Healthcare, Madera, USA

**Keywords:** meningitis, disseminated viral infection, infants, herpes simplex encephalitis, herpes simplex virus

## Abstract

Herpes simplex virus (HSV) encephalitis is one of the most common viral infections in infants associated with high morbidity and mortality rates despite available antiviral therapy. For symptomatic infants, starting empiric therapy with acyclovir can prevent serious neurological sequelae while awaiting results from diagnostic studies. The gold standard of diagnosis remains to be the detection of HSV DNA via polymerase chain reaction (PCR) from cerebrospinal fluid (CSF). However, due to the low viral load in the initial stages of infection, even the gold standard test may not detect active infection. We present a case of an eight-month-old child who presented with fever and seizures and had negative HSV DNA PCR from initial CSF studies. Ongoing fever and recurrent seizures prompted an MRI which was suggestive of meningoencephalitis, HSV DNA PCR from repeat CSF sample resulted positive. This case emphasizes the importance of keen clinical judgment and the caution required when deciding to stop empiric therapy when the clinical suspicion for HSV encephalitis remains high.

## Introduction

Herpes simplex virus (HSV) is a double-stranded DNA virus, belonging to the family of Herpesviridae. Two variants of the virus exist - HSV-1 and HSV-2. HSV-1 is mainly transmitted by oral-to-oral contact and can be transmitted from a mother to an infant easily through contact. HSV-2 is a sexually transmitted infection that causes genital herpes and can also be transmitted vertically through vaginal births [1]. Globally, 67% of people under the age of 50 have HSV-1 and 13% of people worldwide have HSV-2 infection [1]. The majority of infections in adults and older children are asymptomatic and if symptom develops, it includes sores or ulcers around the mouth or genital areas.

However, neonatal and infantile HSV infections are usually severe and have high morbidity and mortality even with treatment. Central nervous system (CNS) infections, such as meningitis or encephalitis, can be caused by both HSV-1 and HSV-2 in the first few weeks of life. Symptoms such as rashes and skin lesions may not be apparent at the time of the exam or reported by caregivers and CNS disease is challenging to diagnose without sharp clinical judgment [2]. Due to the rapidly progressive nature of the disease, it is vital to start empiric therapy with intravenous acyclovir as soon as possible with suspicion to prevent neurological consequences and death. Even with a full course of antiviral therapy, recurrent skin lesions are common in surviving infants [1].

Here, we present the case of an eight-month-old child who initially had seizures along with a fever and an initial negative CSF HSV DNA polymerase chain reaction (PCR). We also identified five additional cases with HSV encephalitis with initial negative CSF HSV PCR.

## Case presentation

An eight-month-old male infant with no significant past medical history presented to the emergency department after his parents noted a fever and multiple episodes of seizure-like activity. The mother denied any complications or infections during pregnancy. No sick contacts or recent infections were reported. His immunizations were up to date. There is no family history of autoimmune disease or seizure disorder. At home, he reached a maximum temperature of 38.8C and his only other observed symptom was “arm twitching” while asleep.

On examination in the emergency department, he had a blood pressure of 94/52, heart rate 175 beats/min, RR 36 breaths/min, temperature 37.5 C, SpO_2_ 98% on room air with weight and height at 56th and sixth percentiles, respectively. He was ill-appearing and irritable. Neurological examination was notable for brisk deep tendon reflexes, symmetric strength in all extremities, and no nuchal rigidity. Initial laboratory investigations revealed a normal electrolyte panel, normal urinalysis, and a complete blood count with a white blood cell count of 17.6 x10^3^/mm^3^ with 58% neutrophils and 33% lymphocytes. C-reactive protein (CRP) was 0.2 mg/dL. Cerebrospinal fluid (CSF) analysis revealed one red blood cell, a white blood cell count of 12 cells/mcL with 15% neutrophils and 58% lymphocytes, and a glucose of 59 mg/dL, and protein of 32 mg/dL. Blood and CSF cultures were sent; the respiratory viral panel was negative. The infant was started on intravenous ceftriaxone and acyclovir and admitted to the acute care floor.

**Figure 1:**
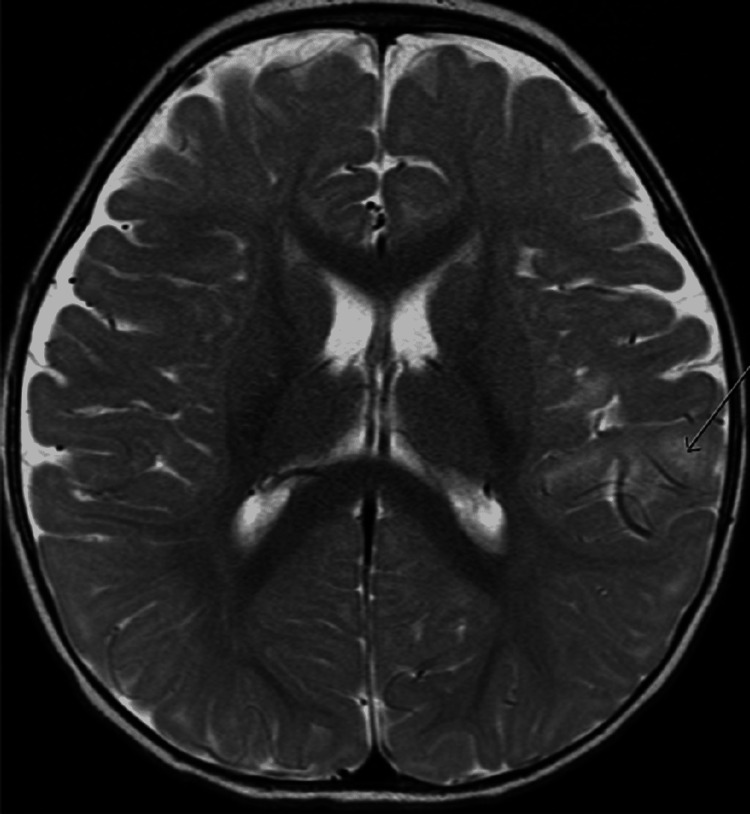
T2 FLAIR post-contrast MRI brain images showing high T2 signal in the posterior left Sylvian cortical region

**Figure 2:**
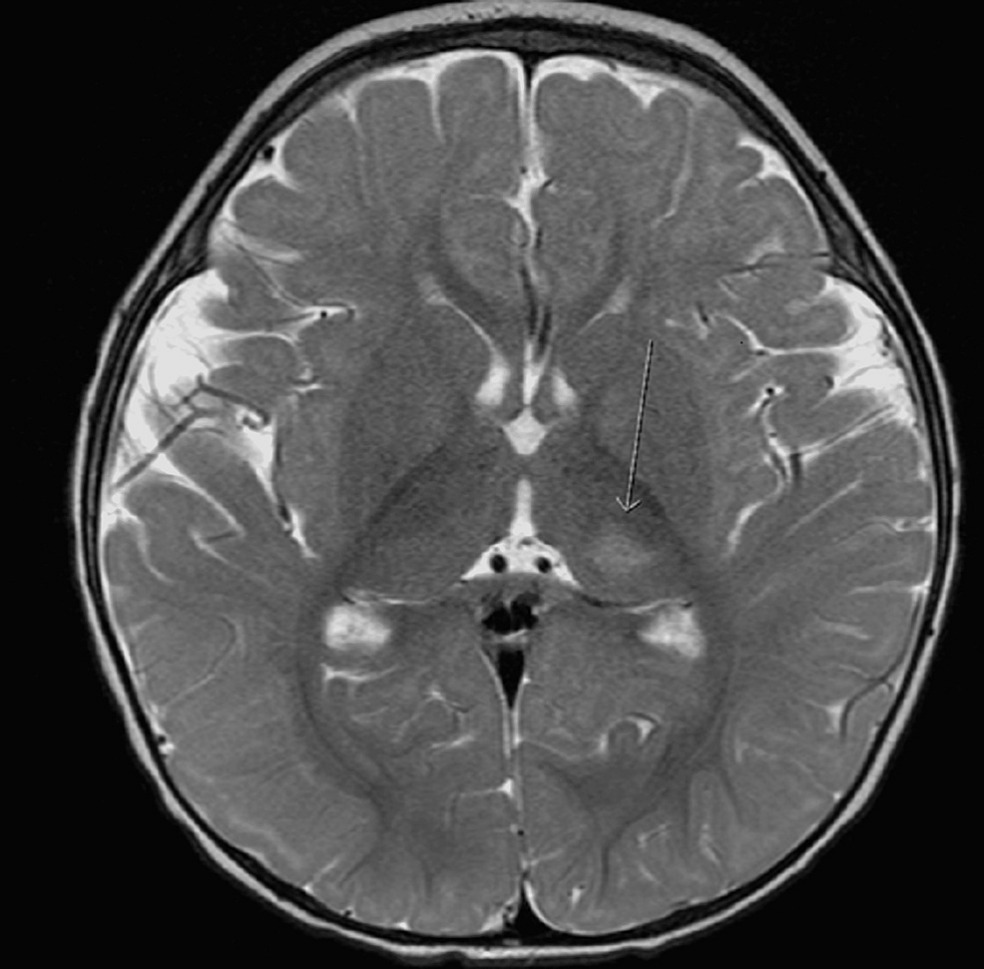
T2 FLAIR post-contrast MRI brain imaging showing high T2 signal in left thalamus.

While admitted, his CSF HSV DNA PCR returned negative and acyclovir was discontinued. Ongoing fever and recurrent seizures prompted magnetic resonance imaging (MRI) of the brain. MRI showed restricted diffusion in the left posterior Sylvian cortical areas concerning meningoencephalitis or vasculitis (Figures 1, 2). Initial coccidioidomycosis screen based on geographic region resulted in negative. Neurology and infectious disease services were consulted who recommended some additional serological studies along with repeat lumbar puncture. Arbovirus panel antibodies, West Nile virus, cytomegalovirus, myeloperoxidase, and serine protease 3 antibodies all resulted in negative.

Lumbar puncture was repeated on the third day of admission due to persistent concern for meningoencephalitis. HSV DNA PCR from repeat CSF revealed positive results for HSV-1. Acyclovir was reinitiated at 20 mg/kg intravenously every eight hours. After sharing the diagnosis with his family, they reported a history of cold sores in the infant earlier that month which was denied during the initial encounter.

The ophthalmology examination was normal. Repeat HSV DNA PCR from CSF obtained on the 20th day of admission resulted in a negative. The child was discharged home after completion of 21 days of therapy with acyclovir with follow-up in the neurology clinic. He passed a hearing evaluation on an outpatient audiology appointment.

## Discussion

HSV is a double-stranded DNA virus. HSV-1 is the leading cause of encephalitis in infants in the United States [3]. Herpes simplex encephalitis (HSE) is a life-threatening condition in infants and is associated with significant morbidity and mortality despite antiviral treatment [4]. Clinical manifestations are hard to attribute to HSE earlier in the illness. These symptoms can include fever, altered mental status, focal neurological deficits, and seizures. It can also present as self-limited aseptic meningitis and rarely as Bell’s palsy [2]. Bilateral temporal lobe abnormalities were once thought to be pathognomonic for HSE but such findings can occur in other diseases [5]. Normal CSF findings have been reported in patients with HSE [6]. HSV DNA PCR from CSF is still the gold standard test for the diagnosis of HSE. The PCR has a sensitivity of 94%-98% and specificity of 98%-100%. The positive predictive value is 95% and the negative predictive value is 98% [7]. The possibility of having a false negative HSV DNA PCR from CSF is very low but not negligible. The initial false-negative results may be a product of the low viral load in the initial stages of infection or contaminants such as red blood cells with hemoglobin in the CSF collection which may interfere with the PCR. Current guidelines by the Infectious Diseases Society of America recommend a repeat CSF HSV DNA PCR within three to seven days while continuing acyclovir therapy if there is a high clinical suspicion of disease in the case of an initial negative result [8]. We have identified five additional cases with HSV encephalitis with an initial negative CSF HSV PCR. There are no published case reports of infants with HSV encephalitis. All cases including our case are summarized in Table 1.

**Table 1:** Summary of Cases of HSV Encephalitis with an Initial False Negative CSF HSV DNA PCR *MRI: Magnetic resonance imaging; CSF HSV DNA PCR: cerebrospinal fluid Herpes Simplex virus deoxyribonucleic acid polymerase chain reaction; GABAAR: Gamma-amino butyric acid type A receptor

Author, Year	Age	Initial Presentation	Initial Diagnostic Studies	Hospital Course	Subsequent Diagnostic Studies	Final Diagnosis	Outcome
Weil et al., 2002 [9]	10 years	Increasing lethargy, fever, aphasia, drooling	Negative CSF HSV DNA PCR* at three separate laboratories	Developed oral apraxia, had a complex partial seizure on Day 3 of hospitalization	MRI* showed multifocal bilateral T2 prolongation with patchy enhancement, repeat CSF HSV DNA PCR* positive on day 4 of admit	HSV encephalitis	Not described
Weil et al., 2002 [9]	37 years	Fever, memory loss, photophobia and disorientation	MRI* showed lateral temporal lobe enhancement, CSF HSV DNA PCR* negative at two laboratories	Not described	CSF HSV DNA PCR* on day ­­­­7 of hospitalization positive	HSV encephalitis	Not described
Weil et al., 2002 [9]	78 years	Fever, disorientation, seizures, coma	MRI* showed temporal lobe edema, negative CSF HSV DNA PCR	Seizures and coma, had a temporal lobectomy	CSF HSV DNA PCR* on day 4 of hospitalization positive	HSV encephalitis	Not described
Adler et al., 2011 [10]	35 years	Altered mental status	Negative CSF HSV DNA PCR*	Discharged home after discontinuing acyclovir therapy, returned with altered mental status	MRI* showed bilateral temporal lobe involvement L>R, second CSF HSV DNA PCR negative	HSV encephalitis	Completed 21 day of acyclovir and returned to baseline
Schuster et al., 2019 [11]	47 years	New onset generalized seizure, behavioral changes	MRI* showed hyperintense FLAIR of left prefrontal gyrus, CSF HSV DNA PCR negative	Acyclovir stopped, methylprednisolone did not improve clinical status	IgG HSV Ab unremarkable. GABA_A_R* Ab detected in serum and CSF. Repeat MRI* showed expansion of FLAIR.	Autopsy revealed extensive HSV encephalitis	Fatal
Devireddy et al., 2022	8 months	New onset seizures, fever	Negative CSF HSV DNA PCR*	Acyclovir stopped, ongoing seizures and fevers	MRI* showed restricted diffusion in left posterior sylvian cortex concerning for meningoencephalitis, repeat CSF HSV DNA PCR* positive	HSV encephalitis	Completed 21 day of acyclovir and returned to baseline upon discharge.

HSE should be considered in infants with fever, seizures, suspicious CSF cell counts, negative bacterial cultures, altered liver function, or coagulopathies. The lack of maternal history of HSV infection does not rule HSV infection as she may be asymptomatic [2]. For patients with high clinical suspicion of HSE, a negative PCR result within the first 72 hours of infection does not completely rule out infection. Even the gold standard test may not be entirely accurate due to the limited viral load present in the initial stages of infection. False-negative HSV PCR results are rare but if HSE is highly suspected through clinical judgment despite an initial negative CSF HSV DNA PCR, a second lumbar puncture and repeat PCR is warranted within three to seven days [8].

Intravenous acyclovir should be started in suspected or confirmed HSE cases as soon as possible. Delay in the initiation of acyclovir has been associated with poor outcomes [12]. Due to the high mortality and devastating neurological outcomes associated with HSE, much caution is needed before discontinuing acyclovir in a patient with concerns for encephalitis. Time spent waiting for definitive diagnosis via HSV DNA PCR from CSF can be crucial for preventing serious sequelae from this disease. Patients with HSE should be treated for 21 days with IV acyclovir. If the significant clinical concern persists even with negative CSF PCR, the recommendation is to complete a course of IV acyclovir for 21 days to minimize the risk. Follow-up with ophthalmology, neurology, and audiology is needed following treatment to identify any neurologic sequelae.

## Conclusions

HSE is a life-threatening condition that can result in vision loss, hearing loss, focal neurological deficits, and ultimately death if left untreated. The acute and rapidly progressive nature of this disease makes it essential to diagnose and treat without delay. If the significant clinical concern persists even with negative HSV CSF PCR, the expert’s recommendation is to complete a 21-day course of IV acyclovir to minimize risk. Decisions on antiviral therapy should not be solely based upon diagnostic results and extreme caution is warranted when deciding to discontinue acyclovir. This case shows us the importance of keen clinical decision-making and the importance of repeat confirmatory studies in the initial stages of disease in a symptomatic child. Even with published guidelines, providers can still miss HSV encephalitis in patients with an initial negative test and may not be aware of the importance of repeat CSF HSV testing in highly suspicious cases.
